# Early onset MSI-H colon cancer with *MLH1 *promoter methylation, is there a genetic predisposition?

**DOI:** 10.1186/1471-2407-10-180

**Published:** 2010-05-05

**Authors:** Eddy HJ van Roon, Marjo van Puijenbroek, Anneke Middeldorp, Ronald van Eijk, Emile J de Meijer, Dianhdra Erasmus, Kim AD Wouters, Manon van Engeland, Jan Oosting, Frederik J Hes, Carli MJ Tops, Tom van Wezel, Judith M Boer, Hans Morreau

**Affiliations:** 1 Department of Pathology, Leiden University Medical Center, Leiden, The Netherlands; 2 Center for Human and Clinical Genetics, Leiden University Medical Center, Leiden, The Netherlands; 3 Department of Pathology, GROW School for Oncology and Developmental Biology, Maastricht University Medical Center, Maastricht, The Netherlands

## Abstract

**Background:**

To investigate the etiology of *MLH1 *promoter methylation in mismatch repair (MMR) mutation-negative early onset MSI-H colon cancer. As this type of colon cancer is associated with high ages, young patients bearing this type of malignancy are rare and could provide additional insight into the etiology of sporadic MSI-H colon cancer.

**Methods:**

We studied a set of 46 MSI-H colon tumors cases with *MLH1 *promoter methylation which was enriched for patients with an age of onset below 50 years (n = 13). Tumors were tested for CIMP marker methylation and mutations linked to methylation: *BRAF, KRAS*, *GADD45A *and the *MLH1 *-93G>A polymorphism. When available, normal colon and leukocyte DNA was tested for *GADD45A *mutations and germline *MLH1 *methylation. SNP array analysis was performed on a subset of tumors.

**Results:**

We identified two cases (33 and 60 years) with *MLH1 *germline promoter methylation. *BRAF *mutations were less frequent in colon cancer patients below 50 years relative to patients above 50 years (p-value: 0.044). CIMP-high was infrequent and related to *BRAF *mutations in patients below 50 years. In comparison with published controls the G>A polymorphism was associated with our cohort. Although similar distribution of the pathogenic A allele was observed in the patients with an age of onset above and below 50 years, the significance for the association was lost for the group under 50 years. *GADD45A *sequencing yielded an unclassified variant. Tumors from both age groups showed infrequent copy number changes and loss-of-heterozygosity.

**Conclusion:**

Somatic or germline *GADD45A *mutations did not explain sporadic MSI-H colon cancer. Although germline *MLH1 *methylation was found in two individuals, locus-specific somatic *MLH1 *hypermethylation explained the majority of sporadic early onset MSI-H colon cancer cases. Our data do not suggest an intrinsic tendency for CpG island hypermethylation in these early onset MSI-H tumors other than through somatic mutation of *BRAF*.

## Background

High frequency of microsatellite instability (MSI-H) is the hallmark of tumors with a mismatch DNA repair (MMR) deficiency. This deficiency leads to an accumulation of somatic mutations, especially in repetitive coding or non-coding DNA sequences (microsatellites) in the genome. MSI-H in colon cancer is found in the context of Lynch syndrome, previously known as hereditary non-polyposis colorectal cancer (HNPCC), in which germline mutations in one of four mismatch repair genes (primarily in *MLH1 *and *MSH2 *[1] and to a lesser extent in *MSH6*[2], *PMS2 *[3] or deletions in *EPCAM/TACSTD1 *(leading to MSH2 methylation) [4,5] are found. Approximately 15% of cases are due to somatic biallelic or hemiallelic DNA methylation of the CpG-rich *MLH1 *promoter sequence, which is associated with gene silencing [6]. Colon cancers with sporadic MSI-H are observed more frequently in females and are often located proximal to the splenic flexure [7].

A clear association between increased age and occurrence of sporadic MSI-H colon cancer was described in 2002 by Young et al. [8]. The combination of age at diagnosis and three pathological features (tumor heterogeneity, peritumoral lymphocytes and tumor-infiltrating lymphocytes) allowed positive identification of 94.5% of MSI-H cancers as either Lynch syndrome or sporadic [8]. As normal aging colon mucosa shows global hypomethylation and specific hypermethylation of tumor associated genes, this epigenetic accumulation can explain the association between sporadic MSI-H colon cancer and older age [9-11]. The rare sporadic cases diagnosed at a relatively young age can provide insight into the etiology of sporadic MSI-H. As young patients with sporadic MSI-H colon cancer are subjected to *MLH1 *methylation without this age-associated epigenetic accumulation, a defect of DNA methylation maintenance or direct targeting of *MLH1 *for methylation could be expected.

*MLH1 *methylation has been one of the hallmarks of the CpG island methylator phenotype (CIMP) since the phenotype was first described in 1999 [12]. The high levels of methylation found in CIMP-high colon tumors suggest that a causative genetic or epigenetic defect influences the spread and initiation of methylation [13]. Somatic *BRAF *mutations, *MLH1 *methylation and sporadic MSI-H are associated with CIMP-positive (CIMP-high and CIMP-low combined) colon tumors in which the bulk of aberrant methylation can be found [13,14]. Aberrant methylation in CIMP-high tumors is thought to arise through an increase in *de novo *methylation. *KRAS *mutations have also been associated with elevated levels of aberrant DNA methylation, although discrepancies between marker panels and techniques showed variable levels of methylation. In general, *KRAS *mutations are associated with CIMP-low (also annotated CIMP2) colon tumors, in which increased levels of aberrant methylation can be detected to some extent, but at lower levels than in the CIMP-high tumors [13,14].

The underlying causes leading to *MLH1 *promoter hypermethylation and subsequently to sporadic MSI-H colon cancer are still largely unknown. A relatively new concept in the field of genetics is germline epimutation. Although rare, multiple studies have described inherited and *de novo *germline methylation of *MLH1 *in patients with Lynch-like colon cancer [15-21]. Cases with confirmed or probable *MLH1 *epimutations are documented to have the same range of tumors as described in Lynch syndrome patients, predominantly early-onset MSI colorectal cancer and endometrial cancer. Although possible, inheritance of the *MLH1 *epimutation is described as very weak, as the *MLH1 *epimutation is unstable in the germline [17,18,20,21]. Paradoxically, patients suspected of having a genetic disorder based on a strong family history may be less likely to carry an epimutation [18]. Germline epimutations are thus highly suspected in young patients presenting with an MSI tumor without a clear family history. Increased risk of MSI-H tumors [22] and tumor-specific *MLH1 *methylation [23] might also be associated with a single-nucleotide polymorphism (SNP) -93 bp from the *MLH1 *transcription start site (rs1800734). This *MLH1 *G>A polymorphism is associated with increased age of onset and CIMP and *BRAF *mutations in individuals with MSI-H tumors [24].

Another possible factor contributing to aberrant DNA methylation is inactivation of *GADD45A *[25], although this finding was later disputed [26,27]. *GADD45A *(growth arrest and DNA-damage inducible protein 45 alpha) is a nuclear protein involved in maintenance of genomic stability, DNA repair and cell growth suppression [28,29]. A recent publication has found *GADD45A *to be a key regulator of active DNA demethylation in *Xenopus *oocytes and cell lines through a DNA repair-induced mechanism [25]. Specific short interfering RNA (siRNA)-mediated knockdown of *GADD45A *and *GADD45B *in the colon cancer cell line RKO induced hypermethylation of *MLH1, THBS1 *and *p16*, three genes known to be involved in carcinogenesis of different types of tumors by DNA methylation [25].

In contrast to MSI-H colon cancer, chromosomal instability (CIN) is enhanced and more pronounced in tumors with a low frequency of microsatellite instability (MSS or MSI-L tumors). This relationship can also be deduced from the observation that MSS/MSI-L tumors often are aneuploid, whereas MSI-H tumors mostly are peri-diploid. Lynch syndrome-associated MSI-H colon cancer hardly shows chromosomal copy number alterations, and the few alterations are mainly restricted to copy neutral LOH (cnLOH) at the mutated locus, especially in *MLH1 *mutated cases [30]. However, sporadic MSI-H colon cancer and MSI-H from patients with unclassified variants in MMR genes seem to show an enhanced (although subtle) number of chromosomal aberrations [30-33].

We studied 46 MSI-H colon tumors showing loss of *MLH1 *expression and its heterodimer *PMS2 *and methylation of the *MLH1 *promoter. Pathogenic germline MMR mutation were excluded. We have primarily focused on comparing relatively young patients with patients of older ages to identify a possible cause for *MLH1 *methylation in young individuals with colon cancer. Tumors were characterized for somatic *BRAF*, *KRAS*, *GADD45A *and the *MLH1 *-93G>A polymorphism (rs1800734), as these genetic factors could play a causative role in *MLH1 *promoter methylation. Whenever material was available, germline *MLH1 *methylation status was studied and DNA sequencing for germline *GADD45A *mutations was performed. In order to analyze whether the younger patients exhibit an intrinsic higher methylation tendency in their genome, the methylation status of eight CIMP markers was determined in the tumors. In a selected subset of young patients, whole genome SNP array analysis was performed on formalin-fixed, paraffin-embedded (FFPE) tumor tissue and compared with previously published data to search for recurrent chromosomal aberrations involved in *MLH1 *methylation.

## Methods

### Patient material

Tumor tissues were obtained from 46 sporadic right sided colon cancer patients analyzed between 1997 and 2006 at the Leiden University Medical Center (Leiden, The Netherlands). MSI analysis, additional MMR immunohistochemistry (IHC) and MMR germline mutation analysis were performed due to a relatively young age of onset and/or a suspected family history of Lynch syndrome. As we mainly focused on comparing relatively young patients with patients of older ages, our sample set was enriched for young patients with *MLH1 *methylation. Our sample set contained a high percentage of *MLH1 *methylated colon cancer patients with an age of onset below 50 years (28%, 13 cases) which is not a reflection the general age distribution of this of type colon cancer. The present study falls under approval by the Medical Ethical Committee of the LUMC (protocol P01-019). Informed consent was obtained according to protocols approved by the LUMC Medical Ethical Committee (02-2004). Patient samples were handled according to the medical ethics guidelines described in the Code Proper Secondary Use of Human Tissue established by the Dutch Federation of Medical Sciences http://www.federa.org.

### DNA isolation and MSI analysis

DNA was isolated from 0.6 mm FFPE punches after assessment of corresponding hematoxylin-eosin stained slides by a pathologist (HM). Standard deparaffination preceded DNA isolation using the Wizard Genomic DNA Purification kit (Promega, Madison, WI, US). The microsatellite instability status of each of the tumors was determined using the Promega MSI analysis system (Version 1.2, Promega, Madison, WI, US) following the recommendations of the National Cancer Institute/ICG-HNPCC [34-36]. Tumors with at least two out of five mononucleotide markers unstable were classified as MSI-H.

### IHC of MMR proteins

Standard three-step, indirect IHC was performed on 4-μm tissue sections that had been transferred to glass slides, including citrate antigen retrieval, blockage of endogenous peroxidase and endogenous avidin-binding activity and di-aminobenzidine development. The following antibodies were used: anti-MLH1 (clone G168-728; BD Biosciences, San Jose, CA), anti-PMS2 (clone A16-4; BD Biosciences), anti-MSH2 (clone GB-12; 1:100; Oncogene Research Products, San Diego, CA) and anti-MSH6 (clone 44; 1:400; BD Biosciences). The utilized secondary antibodies were biotinylated rabbit anti-mouse IgG antibodies (DAKO, Glostrup, Denmark), goat anti-rabbit IgG antibodies (DAKO, Glostrup, Denmark) and biotinylated-peroxidase streptavidin complex (SABC; DAKO, Glostrup, Denmark). Loss of expression was assessed by a complete lack of staining in the tumor cell nuclei with concurrent staining in normal epithelium, stroma or infiltrating leukocytes.

### Mutation analysis

*BRAF *V600E mutations were detected using flanking primers that have been previously described [37]. DNA sequence analysis of codons 12 and 13 of *KRAS *was performed as previously described [38]. For direct sequencing of *GADD45A*, six exon primer pairs were designed (encompassing 100 bp of intronic sequence) using the Primer3 web-tool for the amplification of the four exons of *GADD45A *[39]. The utilized primers are listed in Additional file 1. Primers utilized for sequence analysis of the *MLH1 *-93G>A polymorphism were designed to amplify the region spanning from -231 bp to -51 bp from the *MLH1 *transcription start site [39]. PCR products were purified with the QIAquick PCR Purification kit (Qiagen, Hilden, Germany). Sequencing was performed at the Leiden Genome Technology Center (LGTC, Leiden, The Netherlands) using an ABI 3730 XL (Applied Biosystems, Foster City, CA). Mutational analysis was performed using mutational surveyor (SoftGenetics LLC., State College, PA). Results of all mutational analyses are summarized in Table 1 (extended in Additional file 2).

**Table 1:** Numeric overview: Occurrence of *BRAF *mutations, SNP rs1800734 in relation to age and *MLH1 *methylation status.

		*MLH1 *methylation		*BRAF*	*MLH1*-93G>A			
**Type**	**n**	**M**	**pM**	**Mut**	**G/G**	**G/A**	**A/A**	**NA**

Adenoma	2	2	0	1	0	1	1	0
Carcinoma	44	33	11	24	12	20	7	5
**Age**								

Total <50	13	9	4	4	4	7	2	0
Total ≥ 50	33	26	7	21	8	14	6	5
*BRAF *mut	25	23	2	25	7	12	4	2

### Methylation analysis

Methylation of the 5' regulatory *MLH1 *region at -200 bp (from the transcription start site) was analyzed by using Methylation-Specific PCR (MSP) primers that have been previously described [40]. Sample DNA (100 ng) was mixed with carrier DNA (salmon sperm DNA, 400 ng) followed by bisulfite conversion using the EZ DNA Methylation Gold kit (Zymo Research, Orange, US) and the standard protocol provided by the manufacturer. Amplified fragments were analyzed by electrophoresis through a 2% agarose gel and on an Agilent 2100 Bioanalyzer (Agilent Technologies, Santa Clara, CA). The utilized primers are listed in Additional file 1.

Contamination of the carcinoma tissue by stromal or inflammatory cells was unavoidable in some cases, despite use of micro-dissection, and tumors with a partially methylated phenotype were scored as methylated.

Methylation of MINT1, MINT2, MINT12, MINT31, *RIZ1 *and *TIMP3 *was determined by MSP [14]. Primers and conditions are listed in Additional file 1. MINT27 and Megalin methylation was determined by Combined Bisulfite Restriction Analysis (COBRA) [41]. Tumors were determined to be CIMP-high when four or more markers besides *MLH1 *showed methylation, and tumors were determined to be CIMP-low when containing three or fewer methylated markers besides *MLH1*. For validation of our CIMP marker set, methylation of *IGF2, SOCS1, NEUROG1, RUNX3, CACNA1G *was determined by MSP in the cases with an age of onset less than 50 years [42,43].

### SNP array analysis, copy number changes and loss of heterozygosity (LOH) assessment

Single nucleotide polymorphism array analysis, copy number change and loss of heterozygosity (LOH) assessment were performed as previously described [30]. For each sample, four SNP panels (linkage panel, LP), LP1-4, were tested. All LP panels were combined for testing of the entire genome. LP1 covers chromosomes 1 to 3 and 22, LP2 covers chromosomes 5 to 9, LP3 covers 10 to 15 and 21, and LP4 covers chromosomes 4, 16 to 20, X and Y. Each panel was separately analyzed on a bead array. Due to the limited availability of archival tumor tissue, some of the LPs could not be analyzed. In two cases two LPs and in one case one LP could not be analyzed. To assess the fraction of the genome altered, the number of chromosome cytobands that were altered was divided by the total number of cytobands tested.

### Statistical analysis

Differences in mutation and MSI frequencies between groups were analyzed using Fisher's exact and Chi-Square tests. A p-value below 0.05 was considered to indicate statistical significance. Yates' correction was used whenever a value lower than 5 was used in the Chi-Square test.

## Results

We characterized 13 MSI-H colon cancer cases with *MLH1 *promoter methylation from patients with an age of onset below 50 years and compared these data with those obtained from 33 MSI-H cases of patients over 50 years of age. Based on the Bethesda guidelines, which recommend MSI testing for all colorectal cancers in patients diagnosed before 50 years of age, we used the cutoff age of 50 for our comparisons [36]. The mean age of the study cohort was 61 years (SD is 31 years). The majority of tumors originated from the proximal colon (n = 36, 78% of total), while a low percentage (n = 5, 11%) of MSI-H tumors originated distal from the splenic flexure. All tumors showed loss of expression of nuclear MLH1 and its heterodimer PMS2, confirming the deleterious effect of *MLH1 *promoter methylation. Both MSH2 and MSH6 stained positive and pathogenic germline mutations in any of the four mismatch repair genes were identified in none of the patients.

### Two patients identified with germline MLH1 epimutation

**Figure 1:**
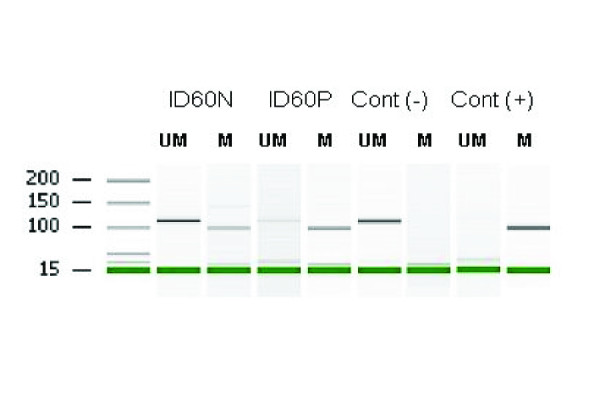
**Lab-on-chip results of a *MLH1 *MSP performed on normal tissue and peripheral blood from patient ID60**. Lane one contains the lab-on-chip DNA marker. Partial methylation of both normal colon mucosa (ID60N) and peripheral blood (ID60P) was observed as they show products produced by the primer pairs amplifying unmethylated (UM) and methylated (M) template DNA. Negative (Neg) and Positive (Pos) controls represent unmethylated (Neg) and methylated (Pos) controls, respectively. The first lane is a visualization of the Agilent DNA 1000 Marker 15/1500 in base pairs (bp)

For seven MSI-H patients with an age of onset below 50 years and 13 patients aged above 50 years, normal colonic epithelium and/or leukocyte DNA was available for germline methylation analysis of *MLH1*. Two female patients were identified as having germline *MLH1 *promoter methylation as both normal colonic epithelium and leukocyte DNA tested positive. The first patient (ID60, Figure 1) presented with a right sided colon cancer at the age of 33 and endometrial cancer at age 52. Her family history showed a sister with endometrial cancer at the age of 37. Apart from a maternal grandfather with colon cancer at the age of 90 years and a maternal niece with duodenum cancer at 39 years, no other tumors from within the Lynch syndrome spectrum were seen. The second patient with germline methylation (ID36) does not have a family history with characteristics of Lynch syndrome. She was diagnosed with colon cancer at age 60 (MSI-H with a *BRAF *V600E somatic mutation) and pancreatic cancer at age 62 (scored as MSS).

### BRAF mutation shows an age-dependent trend in MSI-H tumors

Somatic *BRAF *V600E mutations were found in 25 out of the 44 tested tumors, whereas no *KRAS *codon 12/13 mutations were found (Table 1; extended information in Additional file 2). The majority of the *BRAF *mutations were identified in patients over 50 years of age (n = 21, 65.6% of patients over 50). Comparison with the patient group under 50 years of age (n = 4, 31% of patients under 50) showed a significant difference between the two groups (p-value: 0.044).

### GADD45A somatic and germline DNA mutation analysis

*GADD45A *was successfully sequenced for 38 samples (17 normal epithelium samples and 21 tumor samples). Exon 1 was successfully studied for 37 samples and did not reveal any alterations. We identified 5 cases with an SNP (rs3783466, c.45-23C>T) present in the first intron. Exon 2 was studied for 38 samples and did not reveal any alterations. Exon 3 was sequenced for 38 samples and revealed a variant that was not previously described (in ID70). This heterozygous C>T transition resulted in a neutral amino acid change from proline to serine (p. Pro119Ser) and was predicted by the Sorting Intolerant From Tolerant (SIFT) prediction software to be a tolerated mutation [44]. Exon 4 was studied for 37 samples and did not reveal any alterations.

No association between age, *BRAF *and *GADD45A *mutation status or the *MLH1 *-93G>A polymorphism was observed. An overview of the *GADD45A *mutation data is given in extended information in Additional file 2.

### MLH1 -93G>A polymorphism analysis

We screened 41 out of 46 (13 below 50, 28 above) samples successfully for the *MLH1 *-93G>A polymorphism (rs1800734), by sequence analysis. The G/G genotype was found in 12 samples (29.3%), G/A in 21 (51.2%) and the A/A genotype in 8 (19.5%). No significant differences between tumors grouped by age (with either 50 or 60 years as a cutoff for early onset colon cancer), *BRAF *or *GADD45A *mutational status were observed in our patients. An association between the G>A polymorphism and ages of onset above 50 years (p = 3.5 × 10^-5^) was found in comparison with published control samples (Table 2) [24]. This association was lost (p = 0.19) when comparing younger patients with corresponding published control samples (Table 2) [24]. However, grouping of the A/A and G/A genotypes provided lower p-values when comparing both age groups to controls. A similar distribution of the A allele was found in the young age group as for the patients above 50 years. In this comparison the association between the A allele and the group with an age of onset below 50 years was significant (p = 0.035), although the significance was lost after the required Yates' correction (p = 0.068, Table 2). A numeric overview of the *MLH1 *-93G>A polymorphism sequence data is given in Table 1 (extended information in Additional file 2 and Table 2).

**Table 2:** Genotype frequencies of *MLH1 *-93G>A polymorphism in sporadic MSI-H colon cancer with an age of onset below and above 50 years.

		Genotype frequency (%)					
					
	n	GG	GA	AA	Chi-square	DF	P-value
**Age at diagnosis <50**	13	4(31)	7(54)	2(15)			
							
**Controls 1**	929	554(59.5)	331(35.5)	44(5)	6.0	2	0.19427123*
**Controls 2<60**	501	287(57)	175(35)	39(8)	3.8	2	0.34061584*
							
**Age of diagnosis ≥ 50**	28	8(29)	14(50)	6(21)			
							
**Controls 1**	929	554(59.5)	331(35.5)	44(5)	20.5	2	0.00003502
**Controls 2>60**	1462	883(60)	513(35)	66(5)	22.6	2	0.00001209
		
		**GG**	**GA+AA**		**Chi-square**	**DF**	**P-value**

**Age at diagnosis <50**	13	4(31)	9(69)				
							
**Controls 1**	929	554(60)	375(40)		4.4	1	0.06890141*
**Controls 2<60**	501	287(57)	214(43)		3.6	1	0.10499389*
							
**Age at diagnosis ≥ 50**	28	8(29)	20(71)				
							
**Controls 1**	929	554(60)	375(40)		10.8	1	0.00100409
**Controls 2 >60**	1462	883(60)	579(40)		11.6	1	0.00066844

n: Total number

Chi-square: Value of the chi-square test

DF: Degrees of freedom

Control samples are adapted from literature. Percentages are given in brackets. (Raptis et al., for controls 1 and Samowitz et al. for controls 2 [22,24]). P-values with a * are Yates corrected.

### CpG island DNA methylation is more frequent in older patients and is highly correlated with BRAF mutation in younger colon carcinoma patients

We examined the methylation status of 31 samples (11 below 50 years, 20 above) using six CIMP markers (MINT1, MINT2, MINT12, MINT31, *RIZ1 *and *TIMP3*) with MSP and two CIMP markers (MINT27 and Megalin) with COBRA. Results of the analysis are presented in Additional file 3: Table S1. Although all samples in this sub-selection of our MSI-H study group contain *MLH1 *methylation, a clear age-related trend of methylation was observed. Out of the 11 tested patients that were below 50 years of age and had *MLH1 *methylated colon cancer, only four were shown to be CIMP-high. Remarkably, all of these young CIMP-high cancers showed *BRAF *mutations, whereas such mutations were not detected in samples with less extensive methylation. A higher frequency of CIMP-high (20/20 vs. 4/11, p = 3.1 × 10^-4 ^(Yates' corrected)) was observed for colon cancer patients above the age of 50, concomitant with the higher number of *BRAF *mutations found in these patients.

The methylation status of our early onset cases were validated by use of 5 additional CIMP markers (*IGF2, SOCS1, NEUROG1, RUNX3, CACNA1G*). All validated samples showed similar levels of methylation in both marker sets (Additional file 3: Table S1). Although sample ID1 showed methylation of 3/5 additional markers, the cumulative amount of markers still led us to determine this sample as CIMP-low.

### Genomic profiling of MSI-H colon carcinomas

**Figure 2:**
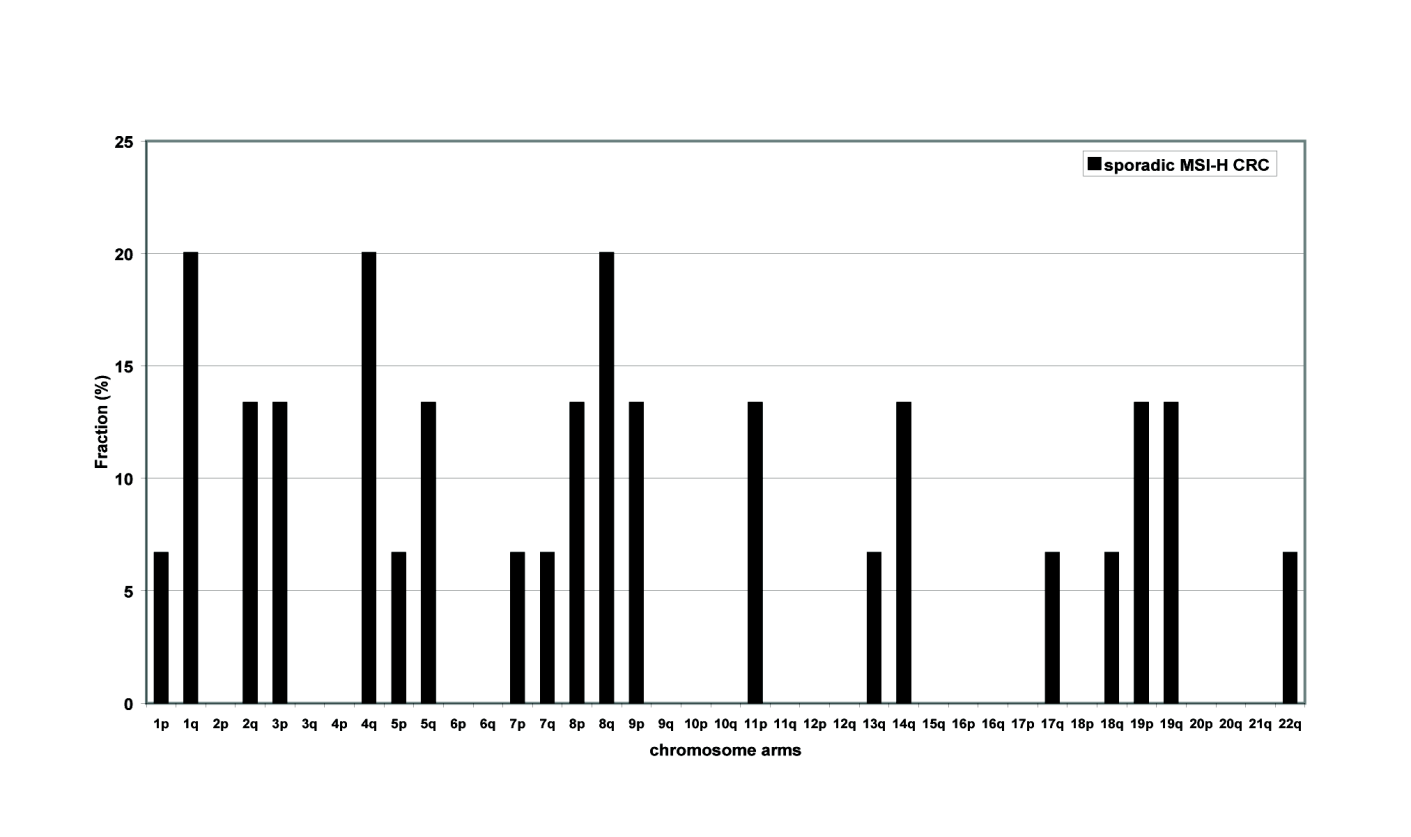
**Chromosomal events per chromosome arm in 15 sporadic MSI-H carcinomas**. Bars indicate the percentage of the tested sporadic MSI-H carcinomas containing a chromosomal aberration or copy neutral LOH per chromosomal arm.

For a sub-selection of 15 MSI-H carcinomas (5 below and 10 above 50 years of age) for which sufficient DNA was available, genome-wide profiles of copy number abnormalities and copy neutral LOHs (cnLOHs) were obtained using SNP arrays suitable for analysis of archival FFPE tissue (Additional file 4: Table S2). Chromosomal copy number changes were observed in 7/15 samples. Physical chromosomal loss was a rare event (on average of 0.2% of the genome) and was only found in 3/15 carcinomas, in which small telomeric regions on chromosomes 1q, 4q, 8p and 18q were deleted. An overview of the events in all tested samples is given in Figure 2. Four chromosomal regions showed cnLOH in more than one tumor: chr 2q23.1-37.3 (n = 2, ID50 and ID59), 3p21.31-26.3 (n = 2; ID18 and ID39, containing *MLH1*), 9p21.2-24.3 (n = 2; ID3 and ID36) and 11p15.1-15.5 (n = 2; ID20 and ID59).

## Discussion

Since CpG island hypermethylation (including *MLH1*) in colon mucosa is considered to be age-related [9], the finding of hypermethylation of *MLH1 *at a younger age is unexpected. Since 2002, several manuscripts pointed to the existence of *MLH1*germline methylation [15-21]. More recently, *MSH2 *methylation due to an inherited deletion in the 3'end of *EPCAM/TACSTD1 *was also discovered [4]. Methylation of *MLH1 *can also be found in addition to a germline MMR mutation, as described by Rahner et al. [45]. We studied 13 MMR germline mutation-negative patients with MSI-H colon cancer (mostly right-sided) at ages of onset under 50 years. These data were compared with those obtained from a control group of 33 patients with an age of onset above 50. The presence of (somatic) promoter methylation of *MLH1 *in the tumors made Lynch syndrome unlikely. We identified two female patients with ages of onset of 33 and 60 years harboring germline *MLH1 *methylation. Relatively young patients without a strong family history who present a MSI-H tumor with loss of MLH1 and PMS2 protein expression are suggested as candidates for *MLH1 *germline epimutation screening [17,21]. We identified one patient with germline *MLH1 *methylation in seven tested cases who were less than 50 years of age, giving a frequency of ~14%. Although the low number of tested samples in this study makes this percentage not representative, this number is not significantly higher than the frequency range of 0.6-13% described in studies screening for germline *MLH1 *methylation in Lynch syndrome-suspected patients [5]. The discovery of germline *MLH1 *methylation in a patient aged 60 years at diagnosis is surprising, as the patients with germline *MLH1 *methylation described prior to this study (n = 25) have a mean age of diagnosis of 37 years with a range of 17-46 [5].

In contrast to the group with an age of onset above 50 years, only some (4/11) of the *MLH1 *methylated MSI-H tumors from patients below 50 years showed high levels of CIMP marker methylation (CIMP-high). For the patient group with an age of onset below 50 years the CIMP-high status completely overlapped with *BRAF *mutations. As both *BRAF *and *KRAS *mutations have been observed in the earliest identified colonic neoplasms, and recent papers have provided evidence that induction of the ras oncogenic pathway will result in DNA hypermethylation, a causative effect of *BRAF/KRAS *mutations is likely [24,46-50]. Instead of widespread CpG island methylation in non-*BRAF *mutated tumors in the early onset patient group, methylation seems to be largely restricted to the *MLH1 *locus. Although the existence of locus-restricted methylation may be a reflection of the Gaussian curve of methylation patterns in relation to age, this finding may suggest a distinct, non-BRAF associated mechanism of *MLH1 *methylation. However, all tumors here were selected upon *MLH1 *promoter methylation which may explain the fact that *MLH1 *is methylated more frequent than all other CIMP genes. As the methylation mechanism is (at least partly) age related, and progressive, a similar selection of tumors methylated on one of the other CIMP markers would have also shown more frequent methylation on these than other CIMP markers including *MLH1 *and occurring in tumors not reaching the CIMP-high classification yet. A progressive methylation and CIMP appearance according to age similar as that shown in Additional file 3: Table S2 favors the argument that *MLH1 *methylation in these young patients is a reflection of the Gaussian curve of methylation patterns in relation to age.

An alternative hypothesis concerning the association between *BRAF *mutation and DNA methylation is that promoter methylation and silencing of specific target genes such as *IGFBP7 *by promoter methylation could favor the selection of activating *BRAF *mutations, since the oncogenic effect of activated BRAF would be enhanced in the absence of IGFBP7's inhibitory function [51]. Since promoter hypermethylation is partly age related the occurrence of *IGFBP7 *hypermethylation and *BRAF *mutation would also explain the diminished occurrence in the young sporadic MSI-H patient group [50]. This role of BRAF in aberrant methylation initiation will have to be elucidated in the future. The locus-specific, non-BRAF associated mechanism of *MLH1 *methylation suggested in our study should be addressed in a larger group of early onset sporadic colon cancer patients with *MLH1 *methylation to provide additional insights.

In patients of older ages, there is an association between somatic *MLH1 *methylation and the *MLH1 *-93G>A polymorphism [22,24,49]. Indeed, when we compared our group of patients above 50 years of age with the published control groups of Raptis et al., and Samowitz et al., we observed an enrichment of the A allele. We explored the possibility that the A allele was more prevalent in the sporadic MSI-H at early ages. However a similar distribution in both age groups was found, no significant enrichment could be found for the cases under 50 years. The hypothesis of Samowitz et al., which suggests an increased likeliness of *MLH1 *methylation in the presence of a CIMP/*BRAF *mutation background and a *MLH1 *-93 G>A polymorphism, excludes young onset patients because of low levels of *BRAF *mutations [24]. Although Samowitz did find a significant difference in A allele distribution between MSI-H colon cancer age groups, our cohort of sporadic MSI-H colon cancer patients with *MLH1 *methylation excluded patients with a germline MMR gene mutation, which might explain the difference found between our studies.

Knockdown and overexpression experiments of *GADD45A *in Xenopus laevis led to the suggestion that deregulation of GADD45A's role in active DNA demethylation could give rise to aberrant methylation [25]. The absence of pathogenic somatic and germline mutations in human *GADD45A *observed in our study and data published during this study [26,27] suggest that a role for *GADD45A *mutations in aberrant hypermethylation in human colon tumors is unlikely.

In a subset of tumors (including five with an age of onset under 50 years), whole genome SNP array analysis of FFPE tumor tissue was used to assess possible causative loci for *MLH1 *methylation. Our copy number and cnLOH analysis identified patterns in agreement with literature describing limited chromosomal instability in sporadic MSI-H colon tumors with *MLH1 *methylation. The extent of copy number abnormalities (CNA) identified here is in agreement with that found by Trautman et al. and by van Puijenbroek et al. [30,33]. In patients under 50 years, no specific genomic pattern was identified, although two cases showed overlapping alterations at chromosome 4q. The smallest region of overlap (region 4q35.1-4q35.2) encompasses the cancer associated genes *TLR3*, *CDKN2AIP*, *ING2*, *CASP3 *and *SORBS2*, none of which are thought to cause aberrant DNA methylation. The four regions of cnLOH that showed infrequent overlap in the 15 tumors tested are not known as such. The cnLOH of 3p21.31-26.3, found in a 44 and a 62 year old, encompasses the 3p22.2 region where *MLH1 *is located. Such cnLOH is not typical for sporadic MSI-H colon carcinomas, but is more readily found in tumors containing pathogenic *MLH1 *mutations [30]. We can not rule out that the identified cnLOH regions may harbor loci involved in *MLH1 *methylation. However, the odds are against such a suggestion.

## Conclusion

Although our study did not identify a cause for *MLH1 *methylation in sporadic MSI-H colon cancer with an age of onset below 50 years, we observed methylation to be almost restricted to the *MLH1 *locus in patients without a *BRAF *mutation. We show that this early onset group consists of two sub-groups: those which are CIMP-high and contain a *BRAF *mutation (resembling sporadic MSI-H in the older age group to a great extend) and those with wild-type *BRAF *and limited methylation in addition to *MLH1 *methylation.

Genomic analysis did not provide recurrent aberrations leading to identification of a possible cause of *MLH1 *methylation in the cases under 50 years. Lastly, we excluded a role for somatic and germline *GADD45A *mutations in the tumorigenesis of early onset sporadic MSI-H colon cancer.

## Competing interests

The authors declare that they have no competing interests.

## Authors' contributions

EHJvR carried out the molecular genetic studies, the sequence alignment, statistical analysis and drafted the manuscript. MvP, DE and EJdM participated in the molecular genetic studies and the sequence alignment. AM and RvE carried out the hybridization of the SNP arrays. JO performed the statistical analysis of the SNP data. FH and CT performed the management of the clinical cases. KADW performed the MSP validation experiments on the additional CIMP markers, under supervision of MvE. TvW, JMB and HM conceived of the study, participated in its design and coordination and helped to draft the manuscript. All authors read and approved the final manuscript.

## Pre-publication history

The pre-publication history for this paper can be accessed here:

http://www.biomedcentral.com/1471-2407/10/180/prepub

## Supplementary Material

Additional file 1 **Primer table**. All primers used in the current study listed. Click here for file

Additional file 2 **Overview of the cohort used**. Gender, age, tumor location, *MLH1 *methylation, *MLH1 *rs1800734, BRAF mutation, KRAS mutation and GADD45A mutation status, when available, are given for each sample used. Click here for file

Additional file 3 Table S1: Overview of the CIMP methylation status Click here for file

Additional file 4 Table S2: Regions of copy number alterations and cnLOH. Click here for file
