# Supplementary material for: Early onset MSI-H colon cancer with MLH1 promoter methylation, is there a genetic predisposition?
Source: BMC Cancer. 2010 May 5;10:180. doi: 10.1186/1471-2407-10-180 (PMC2880297; doi:10.1186/1471-2407-10-180)
Supplement: Additional file 1 — Primer table. All primers used in the current study listed. [file 1471-2407-10-180-S1.PDF]

Primername	Primersequence5'–3'	Annealingtemp	Restriction enzyme	cycles
MLH1_U_fw	TAAAAATGAATTAATAGGAAGAGTGGATAGTG	60		35
MLH1_U_rv	AATCTCTTCATCCCTCCCTAAAACA	60		35
MLH1_MS_fw	AACGAATTAATAGGAAGAGCGGATAGCG	65		35
MLH1_MS_rv	CGTCCCTCCCTAAAACGACTACTACCC	65		35
K-RAS_fw	GACGTTGTAAAACGACGGCCAGTTCGACCCAGGATCCAACTTGCTGAAAATGACTGAATATAAACTTGT	60		33
K-RAS_rv	CAGGAAACAGCTATGACCATGATCCAGTACTTGAGAGAATTCCATCTAGCTGTATCGTCAAGGCACTC	60		33
BRAF_fw	TCATAATGCTTGCTCTGATAGGA	65		33
BRAF_rv	GGCCAAAAATTTAATCAGTGGA	65		33
GADD45a_exon1_fw	TGTAAAACGACGGCCAGTGTGAGTGAGTGCAGAAAGCAG	63		38
GADD45a_exon1_rv	CAGGAAACAGCTATGACCCGCGGGGTAGGTAAGAGAAG	63		38
GADD45a_exon2a_fw	TGTAAAACGACGGCCAGTGTTTTCGTAGAGCCCAGGTG	63		38
GADD45a_exon2a_rv	CAGGAAACAGCTATGACCAGTGATCGTGCGCTGACTC	63		38
GADD45a_exon2b_fw	TGTAAAACGACGGCCAGTGGAGGAAGTGCTCAGCAAAG	63		38
GADD45a_exon2b_rv	CAGGAAACAGCTATGACCGCAGAAAAGCGACAGGAAGT	63		38
GADD45a_exon3a_fw	TGTAAAACGACGGCCAGTGCGCTTCTGCGCTCACT	63		38
GADD45a_exon3a_rv	CAGGAAACAGCTATGACCCGGTCTCCAAGAGCAGGAG	63		38
GADD45a_exon3b_fw	TGTAAAACGACGGCCAGTCTGCGAGAACGACATCAACA	63		38
GADD45a_exon3b_rv	CAGGAAACAGCTATGACCCCTGACTCCCGTCCTTCC	63		38
GADD45a_exon4_fw	TGTAAAACGACGGCCAGTTGTTCAAGTGTTTTCTCCTCAAAAG	63		38
GADD45a_exon4_rv	CAGGAAACAGCTATGACCTGCAATTTGGTTCAGTTATTTTC	63		38
MINT1_U_fw	TATTTTTGAAGTGTTTGTTTGGTGT	57		33
MINT1_U_rv	TCCCTCTCCCCTCTAAACTTC	57		33
MINT1_M_fw	TTCGAAGCGTTTGTTTGG	57		33
MINT1_M_rv	CGCCTAACCTAACGCACA	57		33
MINT2_U_fw	TTTTGTTGAATTTTAGTATTTAAGTTTGT	57		33
MINT2_U_rv	AATAATAACAACAATTCCATACACC	57		33
MINT2_M_fw	CGTCGAATTTTAGTATTTAAGTTCGT	57		33
MINT2_M_rv	AATAATAACGACGATTCCGTACG	57		33
MINT12_U_fw	GGTTTTTTTGTAGATTGTGTTTGTG	57		33
MINT12_U_rv	AAAACATTTTATTTAATTTAAAATCCAAA	57		33
MINT12_M_fw	GTTTTTTCGTAGATTGTGTTTGC	57		33
MINT12_M_rv	CGTTTTATTTAATTTAAAATCCGAA	57		33
MINT31_U_fw	GGGTGGGAATTGAGATGATT	57		33
MINT31_U_rv	CATCACCACCCCTCACTTTA	57		33
MINT31_M_fw	GCGGGAATTGAGACGATT	57		33
MINT31_M_rv	ACGCTTACGCCACTACGA	57		33
RIZ1_U_fw	TGGTGGTTATTGGGTGATGGT	57		33
RIZ1_U_rv	ACTATTTCACCAACCCCAAGA	57		33
RIZ1_M_fw	GTGGTGGTTATTGGGCGACGGC	57		33
RIZ1_M_rv	GCTATTTCGCCGACCCCGACG	57		33
TIMP3_U_fw	TTGAGGATTTAGTGGTAAGTATTGG	57		33
TIMP3_U_rv	ACCAAAAACAAAAAATAACAAAACA	57		33
TIMP3_M_fw	TCGAGGATTTAGCGGTAAGTATC	57		33
TIMP3_M_rv	GAAAACAAAAAATAACGAAACGAA	57		33
MINT27_COB_FW	GGGATTYGGGAAGGTTTTTT	61	TaqI	35
MINT27_COB_RV	ACTCCCTACRACTTAAAACAAAATAC	61	TaqI	35
Megalin_COB_FW	AGTTGGYGGAGGTGTAGATT	55	HhaI	35
Megalin_COB_RV	AACAAACRACRAAAACCAAA	55	HhaI	35
