# Supplementary material for: Early onset MSI-H colon cancer with MLH1 promoter methylation, is there a genetic predisposition?
Source: BMC Cancer. 2010 May 5;10:180. doi: 10.1186/1471-2407-10-180 (PMC2880297; doi:10.1186/1471-2407-10-180)
Supplement: Additional file 2 — Overview of the cohort used. Gender, age, tumor location, MLH1 methylation, MLH1 rs1800734, BRAF mutation, KRAS mutation and GADD45A mutation status, when available, are given for each sample used. [file 1471-2407-10-180-S2.PDF]

ID	Gender	Age	Tumor type	Location	*MLH1* methylation	*MLH1* rs1800734	*BRAF*	*KRAS*	*GADD45A* a Exon_1	*GADD45A* Exon_2A	*GADD45A* Exon_2B	*GADD45A* Exon_3A	*GADD45A* Exon_3B	*GADD45A* Exon_4
1	M	15	N		U				wt	C>T	wt	wt	wt	wt
1	M	15	Ca	NA	pM	G/G	wt	wt						
3	F	27	N		U				NA	wt	wt	wt	wt	wt
3	F	27	Ca	R	Me	G/A	wt	wt						
60	F	33	N	R	Me				NA	NA	NA	NA	NA	NA
60	F	33	Ca	R	Me	G/A	NA	NA						
7	M	36	Ca	L	pM	A/A	wt	wt	wt	C>T	wt	wt	wt	wt
11	M	39	Ca	L	pM	G/A	wt	wt	wt	wt	wt	wt	wt	wt
12	M	41	N		U				wt	wt	NA	wt	wt	wt
12	M	41	Ca	R	Me	G/G	wt	wt						
13	M	42	Ca	R	Me	G/A	V600E	wt	wt	C>T	NA	wt	wt	wt
61	M	42	N		U		wt	wt	NA	NA	NA	NA	NA	NA
61	M	42	Ca	R	Me	A/A	wt	wt						
17	M	43	Ca	R	pM	G/G	wt	wt	wt	wt	wt	wt	wt	wt
18	F	44	N		U				wt	wt	wt	wt	wt	NA
18	F	44	Ca	R	Me	G/A	wt	wt						
20	F	46	N		U				wt	wt	wt	wt	wt	wt
20	F	46	Ca	R	Me	G/A	V600E	wt						
21	M	47	Ca	R	Me	G/A	V600E	wt	wt	wt	wt	wt	wt	wt
23	F	48	N		NA				wt	wt	wt	wt	NA	wt
23	F	48	Ca	R	Me	G/G	V600E	wt						
62	F	50	N		NA				NA	NA	NA	NA	NA	NA
62	F	50	Ca	L	Me		wt	wt						
25	F	52	N		U				wt	wt	wt	wt	wt	wt
25	F	52	Ca	L	Me	G/G	V600E	wt						
27	F	53	Ca	L	pM	G/G	wt	wt	wt	wt	wt	wt	wt	wt
15	M	55	Ca	R	Me	G/A	V600E	wt	wt	wt	wt	wt	wt	wt
29	F	55	Ca	R	pM	G/A	wt	wt	wt	wt	NA	wt	wt	wt
63	M	56	N		U		wt	wt	NA	NA	NA	NA	NA	NA
63	M	56	Ca	R	Me	NA	V600E	wt						
64	M	56	N		U				NA	NA	NA	NA	NA	NA
64	M	56	Ca	R	Me	NA	V600E	wt						
32	M	57	N		U			wt	wt	wt	wt	wt	wt	wt
32	M	57	Ca	R	Me	G/G	V600E	wt						
35	M	59	N		U				wt	wt	wt	wt	wt	wt
35	M	59	Ca	R	Me	G/A	V600E	wt						
36	F	60	N		pM				wt	C>T	wt	wt	wt	wt
36	F	60	Ca	R	Me	G/A	V600E	wt						
37	F	60	Ca	R	Me	G/G	wt	wt	wt	wt	wt	wt	wt	wt
38	F	61	Ca	R	Me	G/A	wt	wt	wt	wt	wt	wt	wt	wt
39	M	62	N		U				wt	wt	wt	wt	wt	wt
39	M	62	Ca	R	Me	G/G	V600E	wt						
42	F	62	N		U				wt	wt	wt	wt	wt	wt
42	F	62	Ca	NA	pM	G/A	wt	wt						
43	F	62	N		U				wt	wt	wt	wt	wt	wt
43	F	62	Ca	R	Me	A/A	V600E	wt						
44	M	64	N		U				wt	wt	wt	wt	wt	wt
44	M	64	Ca	R	Me	NA	wt	wt						
65	F	64	Ca	R	Me	G/G	V600E	wt	wt	wt	wt	wt	wt	wt
47	M	67	N		U				wt	wt	wt	wt	wt	wt
47	M	67	Ca	NA	Me	A/A	V600E	wt						
66	F	69	Ca	R	Me	A/A	V600E	wt	wt	wt	wt	wt	wt	wt
50	M	71	N		U				wt	wt	NA	NA	wt	NA
50	M	71	Ca	NA	pM	NA	NA	wt						
52	F	73	Ca	NA	Me	G/A	V600E	wt	NA	NA	NA	NA	NA	NA
67	M	75	Ca	R	Me	G/A	V600E	wt	wt	wt	wt	wt	wt	wt
68	F	75	Ca	R	pM	G/G	V600E	wt	wt	wt	wt	wt	wt	wt
69	F	76	Ca	R	Me	G/A	wt	wt	wt	wt	wt	wt	wt	wt
54	M	78	Ad	R	Me	G/A	V600E	wt	wt	wt	wt	wt	wt	wt
55	F	78	Ca	R	Me	A/A	V600E	wt	wt	wt	wt	wt	wt	wt
56	F	78	N		NA				NA	NA	NA	NA	NA	NA
56	F	78	Ca	R	pM	G/A	V600E	wt						
57	F	80	N		U				wt	wt	wt	wt	wt	wt
57	F	80	Ca	R	Me	A/A	wt	wt						
70	F	80	Ca	R	Me	G/G	V600E	wt	wt	C>T	wt	wt	C>T	wt
58	F	81	Ca	R	pM	G/A	wt	wt	wt	wt	wt	wt		wt
71	F	83	Ad	R	Me	A/A	wt	wt	wt	wt	wt	wt	wt	wt
59	M	84	N		NA				wt	wt	wt	wt	wt	wt
59	M	84	Ca	R	Me	G/A	V600E	wt						
73	M	90	Ca	R	Me	G/A	V600E	wt	wt	wt	wt	wt	wt	wt

NA: Not available
M: Male
F: Female
N: Normal
Ca: Carcinoma
Ad: Adenoma
R: Right sided
L: Left sided
U: Unmethylated
pM: Partially methylated
Me: Methylated
wt: Wildtype
V600E: *BRAF* V600E mutation
C>T: rs3783466c.45-23C>T
