# Supplementary material for: Early onset MSI-H colon cancer with MLH1 promoter methylation, is there a genetic predisposition?
Source: BMC Cancer. 2010 May 5;10:180. doi: 10.1186/1471-2407-10-180 (PMC2880297; doi:10.1186/1471-2407-10-180)
Supplement: Additional file 3 — Table S1: Overview of the CIMP methylation status [file 1471-2407-10-180-S3.DOC]

**Table S1: Overview of the CIMP methylation status**

| **ID** | **Gender** | **Age** | **Location** | ***BRAF*** | ***GADD45A* mutation** | ***MLH1* rs1800734** | ***MLH1* methylation** | **MIN1** | **MINT2** | **MINT12** | **MINT31** | ***RIZ*** | ***TIMP3*** | **MINT27** | ***Megalin*** | ***IGF2*** | ***SOCS1*** | ***NEUROG1*** | ***RUNX3*** | ***CACNA1G*** | **CIMP** |
| --- | --- | --- | --- | --- | --- | --- | --- | --- | --- | --- | --- | --- | --- | --- | --- | --- | --- | --- | --- | --- | --- |
| 1 | M | 15 | NA | wt | C>T | G/G | pM | M | U | U | U | M | U | M | U | M | M | U | M | U | **Lo** |
| 3 | F | 27 | R | wt | wt | G/A | M | U | U | U | U | M | U | M | U | M | M | U | U | U | **Lo** |
| 7 | M | 36 | L | wt | C>T | A/A | pM | U | U | U | U | U | U | U | U | U | U | U | U | U | **Lo** |
| 11 | M | 39 | L | wt | wt | G/A | pM | M | U | U | U | U | U | U | U | U | M | U | U | U | **Lo** |
| 12 | M | 41 | R | wt | wt | G/G | M | M | U | U | U | M | U | M | U | NA | NA | NA | NA | NA | **Lo** |
| 13 | M | 42 | R | V600E | C>T | G/A | M | M | M | NA | M | M | M | U | M | M | M | M | M | M | **H** |
| 17 | M | 43 | R | wt | wt | G/G | pM | M | U | U | U | M | U | U | U | U | M | U | M | U | **Lo** |
| 18 | F | 44 | R | wt | wt | G/A | M | U | U | U | U | U | U | U | U | U | U | U | M | U | **Lo** |
| 20 | F | 46 | R | V600E | wt | G/A | M | M | M | M | U | U | M | M | M | M | U | M | M | M | **H** |
| 21 | M | 47 | R | V600E | wt | G/A | M | M | M | M | M | M | U | U | M | M | M | M | M | U | **H** |
| 23 | F | 48 | R | V600E | wt | G/G | M | M | M | M | M | U | NA | U | M | M | M | M | M | M | **H** |
| 25 | F | 52 | L | V600E | wt | G/G | M | M | M | M | M | M | M | M | M |  |  |  |  |  | **H** |
| 27 | F | 53 | L | wt | wt | G/G | pM | M | M | U | U | M | M | M | M |  |  |  |  |  | **H** |
| 15 | M | 55 | R | V600E | wt | G/A | pM | M | M | M | U | M | U | U | NA |  |  |  |  |  | **H** |
| 35 | M | 59 | R | V600E | wt | G/A | M | M | M | M | M | M | U | M | M |  |  |  |  |  | **H** |
| 36 | F | 60 | R | V600E | C>T | G/A | M | M | M | M | U | M | M | U | M |  |  |  |  |  | **H** |
| 37 | F | 60 | R | wt | wt | G/G | M | M | M | M | M | M | M | M | M |  |  |  |  |  | **H** |
| 38 | F | 61 | R | wt | wt | G/A | M | M | M | U | M | U | M | U | M |  |  |  |  |  | **H** |
| 39 | M | 62 | R | V600E | wt | G/G | M | M | M | U | M | U | M | U | M |  |  |  |  |  | **H** |
| 42 | F | 62 | NA | wt | wt | G/A | pM | M | M | M | U | M | U | U | U |  |  |  |  |  | **H** |
| 43 | F | 62 | R | V600E | wt | A/A | M | M | M | M | M | M | M | NA | M |  |  |  |  |  | **H** |
| 65 | F | 64 | R | V600E | wt | G/G | M | M | M | M | M | M | M | M | M |  |  |  |  |  | **H** |
| 47 | M | 67 | NA | V600E | wt | A/A | M | M | M | M | U | M | U | U | M |  |  |  |  |  | **H** |
| 66 | F | 69 | R | V600E | wt | A/A | M | M | M | M | M | M | M | M | M |  |  |  |  |  | **H** |
| 67 | M | 75 | R | V600E | wt | G/A | M | M | M | M | M | M | M | M | M |  |  |  |  |  | **H** |
| 68 | F | 75 | R | V600E | wt | G/G | pM | U | M | M | M | M | M | M | M |  |  |  |  |  | **H** |
| 69 | F | 76 | R | wt | wt | G/A | M | M | M | M | M | M | M | M | M |  |  |  |  |  | **H** |
| 56 | F | 78 | R | V600E | NA | G/A | pM | M | M | U | M | M | U | U | M |  |  |  |  |  | **H** |
| 57 | F | 80 | R | wt | wt | A/A | M | M | M | M | M | M | M | M | M |  |  |  |  |  | **H** |
| 70 | F | 80 | R | V600E | C>T | G/G | M | M | M | M | M | M | M | M | M |  |  |  |  |  | **H** |
| 59 | M | 84 | R | V600E | wt | G/A | M | M | M | M | M | M | M | M | M |  |  |  |  |  | **H** |

NA: Not available CIMP: CpG island methylator phenotype

R: Right sided Lo: CIMP-low

L: Left sided H: CIMP-high

wt: Wildtype V600E: *BRAF* V600E mutation

M: Male C>T: rs3783466c.45-23C>T

F: Female

Methylation of markers: MINT1, MINT2, MINT12, MINT31, *RIZ1*, *TIMP3* and Megalin. Available early onset MSI-H colon cancer cases were validated using *IGF2, SOCS1, NEUROG1, RUNX3 and CACNA1G*.
