# Supplementary material for: Early onset MSI-H colon cancer with MLH1 promoter methylation, is there a genetic predisposition?
Source: BMC Cancer. 2010 May 5;10:180. doi: 10.1186/1471-2407-10-180 (PMC2880297; doi:10.1186/1471-2407-10-180)
Supplement: Additional file 4 — Table S2: Regions of copy number alterations and cnLOH. [file 1471-2407-10-180-S4.DOC]

**Table S2: Regions of copy number alterations and cnLOH**

| ID | Gender | Age | *BRAF* | *GADD45A* | CIMP |  |  | **Gain**/*Loss* |  |  | cnLOH |  |  | Missing LP |
| --- | --- | --- | --- | --- | --- | --- | --- | --- | --- | --- | --- | --- | --- | --- |
| 3 | F | 27 | wt | wt | L |  |  |  |  |  | 9p21.2-9p24.3 |  |  | 3, 4 |
| 12 | M | 41 | wt | wt | L |  |  |  |  |  |  |  |  | 4 |
| 18 | F | 44 | wt | wt | L |  |  | **chr:19** |  |  | 3p21.31-3p26.3 |  |  |  |
| 20 | F | 46 | V600E | wt | H |  |  | **8q11.22-8q24.3** |  |  | 1p36.12-1p36.33 |  |  |  |
|  |  |  |  |  |  |  |  | **1q21.2-1q32.2** |  |  | 4q35.1-4q35.2 |  |  |  |
|  |  |  |  |  |  |  |  | *1q42.2-1q44* |  |  | 11p15.1-11p15.5 |  |  |  |
| 23 | F | 48 | V600E | wt | H |  |  | **1q42.13-1q44** |  |  | 17q11.2-17q25.3 |  |  |  |
|  |  |  |  |  |  |  |  | **4q31.21-4q31.23** |  |  |  |  |  |  |
|  |  |  |  |  |  |  |  | *4q32.1-4q35.2* |  |  |  |  |  |  |
|  |  |  |  |  |  |  |  | *18q23* |  |  |  |  |  |  |
| 25 | F | 52 | V600E | wt | H |  |  | **chr:5 ; chr:8** |  |  |  |  |  |  |
| 36 | F | 60 | V600E | C>T | H |  |  | **chr:7 ; chr:14** |  |  | 9p13.2-9p24.3 |  |  |  |
| 39 | M | 62 | V600E | wt | H |  |  |  |  |  | 3p21.32-3p26.3 |  |  |  |
|  |  |  |  |  |  |  |  |  |  |  | 14q31.1-14q32.33 |  |  |  |
| 42 | F | 62 | wt | wt | H |  |  |  |  |  |  |  |  |  |
| 43 | F | 62 | V600E | wt | H |  |  |  |  |  |  |  |  |  |
| 47 | M | 67 | V600E | wt | H |  |  |  |  |  |  |  |  |  |
| 50 | M | 71 | NA | wt | NA |  |  | **chr:19** |  |  | 2q23.1-2q37.3 |  |  |  |
|  |  |  |  |  |  |  |  |  |  |  | 5q35.1-5q35.3  chr:13 |  |  |  |
| 56 | F | 78 | V600E | NA | H |  |  |  |  |  | 5q21.3-5q22.3 |  |  | 3, 4 |
| 57 | F | 80 | wt | wt | H |  |  | **8q24.21-8q24.3**  *8p22-8p23.3* |  |  |  |  |  |  |
| 59 | M | 84 | V600E | wt | H |  |  |  |  |  | 2q14.3-2q37.3  11p12-11p15.5  chr:22 |  |  |  |

F: Female NA: Not available

M: Male LP: Linkage panel

L: CIMP-low V600E: *BRAF* V600E mutation

H: CIMP-high C>T: rs3783466c.45-23C>T

wt: Wildtype
